# Cancers in Vietnam—Burden and Control Efforts: A Narrative Scoping Review

**DOI:** 10.1177/1073274819863802

**Published:** 2019-07-18

**Authors:** Tung Pham, Linh Bui, Giang Kim, Dong Hoang, Thuan Tran, Minh Hoang

**Affiliations:** 1 Department of Physiology, Hanoi Medical University, Hanoi, Vietnam; 2 Center for Population Health Science, Hanoi University of Public Health, Hanoi, Vietnam; 3 Department of Health Education, Hanoi Medical University, Hanoi, Vietnam; 4 Vietnam National Cancer Institute, National Cancer Hospital, Hanoi, Vietnam; 5 Department of Health Economics, Hanoi University of Public Health, Hanoi, Vietnam

**Keywords:** cancer, cancer control, cancer burden, prevention, early detection, screening, treatment, health systems, health policy, review, NCDs, Vietnam

## Abstract

Although the burden of cancer is rapidly growing in Vietnam, there was no up-to-date review that describes cancer burden and control in Vietnam throughout the literature. By identifying various risk factors, means of prevention, and methods for early detection, this review seeks to systematically summarize the evidence for the future planning and management of cancer occurrence in Vietnam. Additionally, this report aims to identify improvements which are necessary for the treatment and palliative care of patients with cancer in Vietnam. We employed a hybrid approach including both a scoping review and narrative synthesis for this study. Information was identified, extracted, and charted from various sources, which include international and domestically published studies, in addition to gray literature. Our results illustrate that the burden of cancer in Vietnam has tripled in the past 30 years, and this situation could be partly explained by the growing prevalence of both old and new risk factors. Besides hepatitis B virus, various other important risk factors such as human papilloma virus, tobacco usage, physical inactivity, and improper diets are still not under control in Vietnam. There is presently a lack of national cancer screening programs, and the capacity of cancer care services could not maintain pace with the demands of a rapidly increasing Vietnamese population. Overall, policy frameworks for cancer control in Vietnam are in place, but there is still a lack of proper financing and governing models necessary to support a sustainable program. In conclusion, Cancer and its associated consequences are both persistent and emerging problems in Vietnam, and the results of cancer control programs are limited. A comprehensive and evidence-based approach toward the prevention and treatment of cancer should be the future direction for Vietnam.

## Introduction

The International Agency for Research on Cancer (IARC) estimates that in 2018, there would be 18.1 million new cases and 9.6 million deaths linked to cancer worldwide.^1^ Unfortunately, the burden of cancer is disproportionate among nations, in which developing countries account for 57% of cases and 65% of deaths associated with cancer.^2^ However, these countries, including Vietnam, only received about 5% of the global cancer financial resources.^3,4^

In Vietnam, the estimated number of new cases in 2018 was 164 671 (0.17% of the population) and the estimated number of deaths in 2018 was 114 871 (0.12% of the population).^1,5^ Both of these statistics have tripled in the past 30 years.^6^ Nevertheless, the quality of national cancer registry data that were used as an input to calculate the statistics above has numerous deficiencies. For example, there is a lack of national data that describe both cancer survivorship and mortality.^7,8^

Although Vietnam proposed the National Cancer Control Programme (NCCP) in 2002,^9^ implemented the NCCP in 2008,^7^ and reasserted the commitment to this program in the national strategy for the prevention and control of noncommunicable diseases (NCDs),^10^ reports regarding cancer burden and control are still not well-documented in the literature. Therefore, to elucidate both the burden and control effort of cancer in Vietnam, we conducted a narrative review to summarize update-to-date evidence for the future planning and management of various risk factors, implementing effective prevention and early detection programs as well as the improvement in treatment and palliative care for patients with cancer in Vietnam.

## Objectives

The primary objective of this article is to summarize and discuss both the burden of cancer and control efforts of this disease in Vietnam. We aimed to review available articles and gray literature on cancer burden and control. In addition, we identified issues for discussion which are necessary to strengthen health systems while disseminating new areas of interest for future research.

## Methods

In this article, we used a hybrid of scoping review and narrative synthesis approach.^11^ Since there is a lack of review on cancer in Vietnam and the scope of the research question was quite broad, a scoping review in tandem with a narrative synthesis was deemed to be the most appropriate methodology for this study.^11^ The scoping review design would allow us to identify broad and diverse findings in numerous disciplines. A narrative synthesis would provide a context which would describe how it is necessary for different stakeholders and components to work together for a sustainable health system in Vietnam. The presented information for this study was identified, extracted, and charted from various sources, including international and domestically published studies, as well as gray literature. The review process and final review paper were prepared in accordance with the guidelines on scoping reviews as published by Peters et al^12^ and the Preferred Reporting Items for Systematic Reviews and Meta-Analyses (PRISMA) statement^13^ (Supplemental PRISMA checklist).

We conducted a systematic search for peer-reviewed articles in the English language which were indexed in the MEDLINE database in October 2018. The present search strategy was developed to include studies with the following terms in the title/abstract: “Vietnam” OR “Viet Nam” AND “Cancer” together with “Burden” OR “Control” OR “Prevention” OR “Risk factors” OR “Early detection” OR “Screening” OR “Diagnosis” OR “Treatment” OR “Palliative care.” Eligible articles for this study were publications that reviewed or reported empirical data in relation to the previously mentioned topics in Vietnam. Papers that reported findings in Vietnamese from the context of other countries (eg, Vietnamese American) were excluded.

To identify related studies that were published domestically, we also searched the electronic databases at Hanoi Medical University Library, Hanoi University of Public Health Library, and *Vietnam Oncology Journal* collections at the National Cancer Hospital in October 2018. The same criteria that were noted above and the equivalent keywords in the Vietnamese language (gánh nặng ung thư, kiểm soát ung thư, phòng chống ung thư, dự phòng ung thư, chẩn đoán sớm ung thư, sàng lọc ung thư, tầm soát ung thư, chăm sóc giảm nhẹ ung thư, nguy cơ ung thư) were used in our search strategy.

Relevant gray literature was identified through an online search by employing the search engine Google, in addition to the websites of Vietnamese governmental agencies, international organizations, and nongovernmental organizations in Vietnam.

Key findings from documents in the Vietnamese language were summarized and translated into English. All relevant details from these documents, including titles, the year of publication, significant findings, and so forth, were extracted and charted using a standardized data extraction spreadsheet, which was provided by The University of Texas Health Science Center at Houston–School of Public Health Library,^14^ for further analysis. Using the data extraction sheets, we summarized information from the included documents and identified discrepancies and gaps in Vietnam’s cancer control effort. This process was completed by comparing recommendations from both the World Health Organization (WHO) and best practices initiatives in cancer prevention worldwide with the results from our review. Based on such information, we sought to recommend appropriate policy actions that could leverage Vietnam’s NCCP in the near future.

## Results

**Figure 1.**
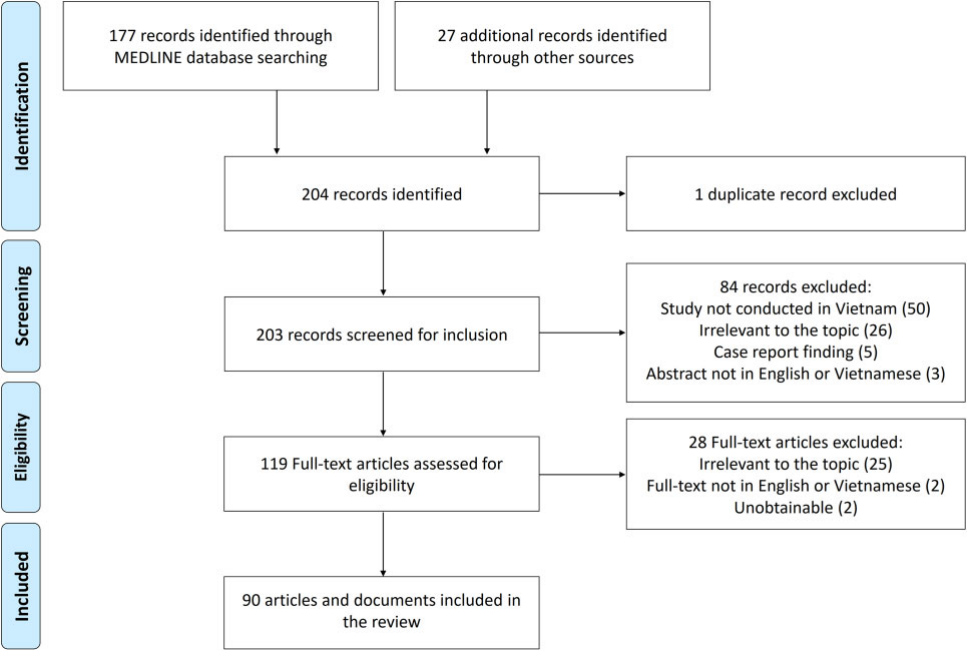
PRISMA flowchart showing the selection process of articles.

Our search strategy identified 177 English articles and 11 Vietnamese articles/research reports that met the inclusion criteria for this study. We also included 13 gray literature documents with information from international organizations and nongovernmental organizations in our review. Three more articles or documents were identified through bibliographies screening (Figure 1 and Supplemental Table 1). Our literature search did not identify any reviews that detailed this topic.

### Burden of Cancer

To estimate the burden of cancer, Vietnam established its first cancer registry in Hanoi in 1984.^7^ Since then, 6 cancer registries were established in Hanoi, Hai Phong, Thai Nguyen, Ho Chi Minh, and Can Tho until the year 2010,^15^ and 3 more were recently developed to improve the geographical representation of these registries.^7^ The Vietnam National Cancer Institute reported that most of the statistics acquired as inputs for their database were hospital based, and the quality of data varied among various regions in Vietnam.^7^ The Global Initiative for Cancer Registry Development (GICR) Partners Task Force reported that the Hanoi and Ho Chi Minh City registry had the best overall practices and data quality in the country which covers 45 and 30 hospitals, respectively, in their catchment areas.^16^

**Figure 2.**
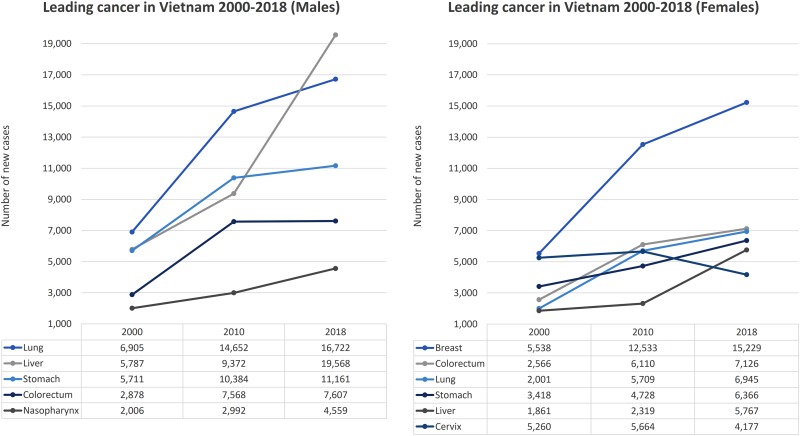
Trend of leading cancer incidence in Vietnam, 2000 to 2018.

Based on both the Vietnamese cancer registry data and mathematical modeling, the IARC estimated a total of 164 671 new cases and 114 871 cancer deaths occurred in Vietnam during 2018.^1,5^ Both of these statistics had tripled since 1990, when there were 52 700 new cases and 37 700 deaths associated with cancer.^6^ The IARC also stated in the 2018 report that there were 300 033 people currently living with cancer in Vietnam (current 5-year prevalent cases).^5^ A detailed overview regarding the incidence, prevalence, and mortality of all cancer types can be retrieved from the Global Cancer Observatory–Vietnam Population fact sheets 2018.^5^ Regardless of sex, liver cancer remained the most common cancer and the number one killer of patients with cancer (25 335 new cases; 25 404 deaths), followed by lung (23 667 new cases; 20 710 deaths), stomach (17 527 new cases; 15 065 deaths), and breast (15 229 cases; 6103 deaths).^5^ The current leading cancers were the liver, lung, stomach, colorectum, and nasopharynx in males, while cancers of the breast, colorectum, lung, stomach, and liver were more typical in females^5^ (Table 1). The ranking of common cancers in males seems to be stable with the domination of the liver, lung, and stomach cancers^5,17,18^ (Figure 2). In females, breast, colorectum, and lung cancers were the most common types observed in recent years (2010-2018).^5,17^ Although the overall cancer incidence was on the rise, the national registry data suggest that the incidence of cervical and oral cancer is in decline.^5,17,19,20^

**Table 1.** Leading Cancer Incidence in Vietnam, 2000 to 2018.

Rank	Males			Females		
	2000^a^	2010^a^	2018^b^	2000^a^	2010^a^	2018^b^
1	Lung	Lung	Liver	Breast	Breast	Breast
2	Liver	Stomach	Lung	Cervix	Colorectum	Colorectum
3	Stomach	Liver	Stomach	Stomach	Lung	Lung
4	Colorectum	Colorectum	Colorectum	Colorectum	Cervix	Stomach
5	Nasopharynx	Esophagus	Nasopharynx	Lung	Stomach	Liver

^a^ Nguyen Ba Duc, et al.^17^

^b^ International Agency for Research on Cancer (IARC).^5^

The Vietnam Burden of Disease and Injury Study in 2008 estimated that cancer was responsible for 22% of the total years of life lost (YLL)—nearly 1.5 million years—and ranked only lower than cardiovascular diseases (27% of total YLL).^21,22^ The same study also calculated that cancers ranked third among the leading causes of disease burden in Vietnam, which accounted for 14% of total disability-adjusted life years (DALYs)—nearly 1.7 million years.^21,22^ One article also suggested that colorectal cancer in Vietnam seems to appear at an earlier age in relation to the Asia Pacific consensus for recommended screening age—50 years old.^23^ The proportion of early-onset (younger than 50 years) colorectal cancer cases in this study was 28%.^23^

Among all patients with cancer, only 20% to 30% of cases appeared at early stages,^7,9^ and 1-year incidence of mortality was up to 25%.^24^ Other studies also revealed that the 5-year survival rate of patients with breast cancer in Vietnam was lower when compared to other countries with similar distributions at the stage of diagnosis.^25^ In addition, Vietnamese children with lymphoblastic leukemia also experienced poor relapse-free survival rates compared to Caucasian children.^26^

The cost of cancer treatment in Vietnam is dependent on the stage of diagnosis, access to public or private hospitals, and range of treatment options (US$11.7-US$11 400, regarding breast or cervical cancer).^7,27,28^ The ACTION (ASEAN Costs in Oncology) prospective cohort study reported that, in Vietnam, the 1-year incidence of financial catastrophe was 68%, which was the highest in the ASEAN region.^24^ This study identified that 37.4% of the households with patients with cancer were pushed into significant poverty due to high treatment costs.^29^

A cross-sectional study on the quality of life among patients with breast cancer using the European Organization for the Research and Treatment of Cancer Quality of Life Questionnaire C30 illustrated that the mean global health status score of these patients was estimated to be 58.6 of 100 points.^30^ The authors also identified several factors that were associated with quality of life, such as age, level of education, body mass index, treatment, and the stage of cancer.^30^ Patients with cancer also experience other comorbidities that can be associated with their disease, such as depression. The overall prevalence of serious depression among patients with cancer in Vietnam was 28%, which is 10 to 15 times higher than the general population relative to studies conducted in the same region.^31^

### Important Risk Factors

#### Hereditary and genetic factors

Our review identified 4 studies that assessed several hereditary and genetic risk factors in the Vietnamese population. These studies include GSTA1 genotype and gastric cancer;^32^ hsa-miR-122 and hepatitis B virus (HBV)–related hepatocellular carcinoma (HCC),^33^ *RB1* gene mutation, and retinoblastoma.^34^ The fourth study reported an association between family history and an increased risk of early-onset colorectal cancer among patients in Ho Chi Minh City.^23^ The Vietnam National Cancer Institute also stated that hereditary and genetic risk factors for cancer onset were not well-documented in Vietnam due to the lack of research capability in cancer biology.^7^

#### Infection

Infection with HBV and infection with human papilloma virus (HPV) remain significant risk factors for cancer incidence in Vietnam. Although universal HBV vaccination of infants is presently in effect in Vietnam, HBV-related liver disease burden was expected to increase.^35^ The chronic HBV prevalence in Vietnam grew from 6.4 million cases in 1990 to 8.4 million in 2005 and was expected to decrease to 8.0 million by 2025.^35^ However, HBV-related HCC incidence was predicted to increase from 9400 in 1990 to 25 000 in 2025.^35^ Another cross-sectional study illustrated that 68.2% of participants in the rural areas of Vietnam had evidence of HBV exposure.^36^ Risk factors for HBV identified by this study were being 60 years of age or older, hospital admissions, history of acupuncture, household contact with a person living with liver disease, reusing syringes, and sharing razors.^36^

In Vietnam, the HPV vaccine was not included in the national vaccination program at the time of this review, and individuals are obligated to pay out of pocket for the vaccine. The National Action Plan on Prevention and Control of Cervical Cancer set a goal that at least 25% of all girls and women would receive the HPV vaccine by 2025.^37^ Among the Vietnamese population, HPV DNA was detected in 97% cases of invasive cervical cancer, and the most common types were HPV 16 (50%), HPV 18 (35.4%), and HPV 52 (6.2%).^38^ Other cross-sectional studies and reviews reported that the prevalence of HPV infections among Vietnamese women ranged from 3.1% to 10.9% depending on the location of the study, and HPV type 16 and 18 were also the most common followed by type 58.^39-45^ In many provinces such as Hanoi and Can Tho, approximately 90% of the infection cases were caused by high-risk HPV strains (types 16, 18, 31, 33, 35, 39, 45, 51, 52, 53, 56, 58, 59, 66, 68, and 82).^40,41^ Human papilloma virus infection was associated with sexual habits, in addition to the presence of herpes simplex virus 2 antibodies, early age at one’s first sexual encounter, the number of lifetime sexual partners, history of childbirth, and usage of oral contraceptives.^43,44^ In a study by Vu and colleagues, the group found that only about one-third of women have ever heard about HPV, the HPV vaccine, and were aware that HPV is a risk factor for cervical cancer.^39^ The level of awareness of participants in this study also differed vastly across geographical areas.^39^

Regarding other infections in relation to cancer, the prevalence of Kaposi sarcoma–associated herpesvirus (KSHV) among women in Hanoi was around 11.3% and in Ho Chi Minh City was 15.5%.^46^ Unfortunately, this cross-sectional study did not test for HIV infection among the participants in the previously mentioned regions.^46^ In this study, the prevalence of KSHV was slightly increased in patients with a higher age in areas with a high prevalence of KSHV and it decreased in patients who have attained a higher degree of education.^46^ Moreover, *Opisthorchis viverrini* and *Clonorchis sinensis* liver flukes, which were linked to cholangiocarcinoma and HCC by the IARC,^47,48^ are also common in Vietnam with nearly a million cases.^47^ *Clonorchis sinensis* is widespread in northern Vietnam (prevalence ranged from 5% to 26%), while *O viverrini* is endemic in the central and southern Vietnam.^47^ Both of these parasites were found to be linked to the habit of eating raw or undercooked fish.^47^

#### Behavioral risk factors

In the Vietnamese male population, smoking was responsible for 28% of all-cause deaths in adults and 85% of lung cancer death.^49,50^ The prevalence of tobacco smoking in males was reduced from 56.1% (2002)^7,10,51^ to 47.4% (2010)^7,10,51^ and to 45.3% (2015).^7,10,51^ However, this reduction was not as high as expected.^51^ Exposure to secondhand smoking is notably high and ranges from 48% to 70% in homes and outdoor or public places.^52^

Other case–control studies in the Vietnamese population identified that alcohol use and physical inactivity were linked to an increased risk for breast cancer (odds ratio [OR]: 1.85 and 2.2, respectively).^53,54^ Diets high in carotenoids were associated with a decreased prostate cancer risk (OR for lycopene: 0.46, tomatoes: 0.39, and carrots: 0.35).^55^ Conversely, roasted meats, bread, and biscuit consumption were associated with increased stomach and colorectal cancer risk (OR: 1.63, 1.4, and 1.6, respectively).^56^ In 2015, the Vietnam national survey on the risk factors of NCDs (STEPS) in 2015 estimated that 77.3% of males and 11% of females were current alcohol drinkers (43.8% overall) and that there was an increasing trend in alcohol comsumption.^57^ The STEPS survey also reported that the prevalence of insufficient physical activities was 20.2% in men and 35.7% in women (28.1% overall).^57^ A recent review of NCDs and risk factors pointed out a distinct change in Vietnamese lifestyle, in which the general population changed their dietary pattern by increasing the intake of both foods with rich quantities of proteins and fats between the years 1981 and 2010.^52^ This review and the STEPS survey also indicate that there is a high consumption of salt (9.4 to 22 g/person/d), sugar-sweetened beverages (more than 900 million liters/year for the whole population), and low consumption of fruit and vegetables (57.2%-80% of the population with less than 5 serving/day) in typical diets.^52,57^ Other hazard factors such as betel quid chewing, which was associated with an increased risk of oral cancer, was shown to be in decline among the general Vietnamese population.^58^

Regarding other physiological and metabolic risk factors, we did not identify any study that assessed the association of these risk factors and cancer in Vietnam. However, the Vietnamese population is presently experiencing an increased prevalence of hypertension (15% in 2002 to 20% in 2015) and overweight/obesity (2.3% in 1993 to 15% in 2015) among adults.^52,57^ In addition, the prevalence of diabetes mellitus and elevated blood cholesterol among adults in 2015 was 4.1% and 32%, respectively.^52,57^

#### Environmental risk factors

We identified 4 case–control studies that assessed several environmental risk factors that are involved with cancer risk. The most recent article, which was published in 2017, collected data from 195 patients with breast cancer and 254 controls; the authors reported that elevated blood cadmium level was associated with increased breast cancer risk.^59^ In another case–control study with 152 male cases of HCC and 241 controls, the authors identified an association between the use of organophosphate-based pesticides and increased HCC risk.^60^ However, 2 smaller studies, which included only 21 and 87 cancer cases, did not find the association between organochlorines with choriocarcinoma and breast cancer.^61,62^

### Screening and Early Detection Programs

One of the first recorded screening programs that we identified for this study was for cervical cancer. In 1996, the burden of cervical cancer in Vietnam was shown to be associated with troop movements during the Vietnam War.^63^ In general, women whose husbands were enrolled in military services presented an increased risk for cervical cancer, though this was dependent on the geographical areas (OR: 2.6-3.9).^63^ Based on that finding, the Viet/American cervical cancer prevention project established a Papanicolaou screening service and recorded a decrease in cervical cancer incidence from 29.2 in 100 000 in 1998 to 16 in 100 000 in 2003.^64^ Presently, there is still a lack of national screening and early detection programs, which are available to the entire population. Limited resources only allow for some pilot screening programs to be conducted on a small scale, which covers about 2% of the target population.^8^ The shortage of trained personnel, lack of appropriate diagnostic equipment, and the lack of appropriate funding mechanisms from the national health insurance are major factors which prevent the scale-up of cancer screening in Vietnam.^65^ Since there is no nationwide screening program, Vietnamese people are expected to actively seek screening services at health-care units independently without reimbursement from the national health insurance system.^65^ A cross-sectional study on female workers in Ho Chi Minh City in 2016 observed that 35.2% of the participants never had a Pap smear test, and only 28.3% reported having a Pap smear test within the past 3 years.^66^ The STEPS survey also indicated that only 24.9% of women between the ages of 18 and 69 and 31.5% of those aged 30 to 49 had ever had cervical cancer screening.^57^

From 2008 to 2015, pilot screening programs for cervical, breast, oral, and colorectal cancers^65^ were implemented with the support of various domestic and international partners.^7,8,67,68^ During the period between 2008 and 2010 alone, nearly 100 000 women aged 30 to 54 years received cervical cancer (with Pap smear test) and breast cancer (with breast examination) screenings, and 9634 individuals received oral cancer (with visual inspection) and colorectal cancer (with Fecal Occult Blood Test) screenings throughout Vietnam.^7,8,67^ Support from the International Atomic Energy Agency in 2015 to 2016 allowed for 24 000 more women in Hanoi and Can Tho access to cervical and breast cancer screening services by employing both visual inspections with acetic acid (VIA) techniques and breast examinations.^7^ The Vietnam National Cancer Institute reported that the most significant drawbacks of the previously mentioned screening programs were the cost and technical difficulties of subsequence follow-up; this is a limitation as health-care teams from central/provincial hospitals had to come to their local communities to organize such programs regularly.^7^ Despite these difficulties, cervical cancer screening programs in Vietnam and other developing countries showed that VIA and cryotherapy is a feasible approach to identify cancer cases in low-resource settings.^69^ Screenings for prostate cancer using prostate-specific antigen tests were also considered, but the benefits of such mass screening programs have not been proven.^70^

### Diagnosis and Treatment Capacity

At the time of this review, the health-care services in Vietnam can be described as disease-specific and vertically organized.^71^ Most of the cancer treatments were provided at provincial or national hospitals, and the 2 largest national cancer centers are “K Hospital”—The National Cancer Hospital, located in Hanoi, and Ho Chi Minh City Cancer Hospital, located in Ho Chi Minh City. In 2016, there were 3 comprehensive cancer centers and 45 oncology departments located at general hospitals throughout Vietnam.^7^ The Vietnam Ministry of Health recommends that cancer surgery should be conducted at provincial/national hospitals, but benign tumors could be surgically removed at district-level hospitals.^72^ However, a report from 2010 illustrated that 10 of 63 provincial hospitals throughout Vietnam were unable to provide services for patients with cancer.^15^ The number of health staff with sufficient knowledge and skills related to the treatment of cancer was also inadequate.^65^ With the current amount of limited resources, the present health-care system could only meet 30% to 40% of the demand for cancer services in Vietnam.^65^

The most significant problem regarding cancer diagnosis in Vietnam and many other developing countries is access to quality pathology services. Currently, the Vietnamese health-care system has only recorded pathological results from 48% of patients, who were diagnosed with cancer and/or received cancer treatment,^68^ and 9 of 63 provincial hospitals in Vietnam lack a pathology department.^15^ Many modern techniques for immunohistochemistry and molecular analysis are only available at a small number of comprehensive cancer centers,^7^ and the accuracy of pathology testing at the centers that host these facilities is still limited.^7^ A recent report from a team of hematopathologists at the University of Minnesota Medical School demonstrated that the exact/complete diagnostic concordance in comparison to their tests was 50% or less for all 3 hospitals that the team visited while in Vietnam.^73^

Regarding the accessibility of radiation therapy, the Vietnam National Cancer Institute reported that in 2016, there were 36 linear accelerators (an increase from 13 linear accelerators in 2010), which could deliver 3-dimensional conformal radiation therapy, in Vietnam.^7^ In addition, it was noted that advanced techniques, such as intensity-modulated radiation therapy, were limited in Vietnam.^7^ In several cancer centers, older radiation techniques such as cobalt-60 external radiotherapy machines were still in use due to its low cost.^7^ Nevertheless, the 2016 report of Vietnam National Cancer Institute stated that many provinces did not have radiotherapy treatment facilities.^7^

In terms of accessibility to cancer drugs, the QuintilesIMS Institute reported that 42 new cancer drugs were launched during the period 2011 to 2015, but only one of these drugs was available in Vietnam’s pharmaceutical market.^74^ This fact is not only limited to cancer drugs as several essential drugs for NCDs management were also found to be unavailable for distribution at community health stations.^65^

Although 70% to 80% of Vietnamese patients with cancer were diagnosed at the terminal stage,^7,8^ palliative care units only exist in Hanoi, Ho Chi Minh City, and 3 other provincial hospitals.^7^ There is also a complete lack of hospice care service for patients with cancer in Vietnam.^7^ On the other hand, opioids for pain relief have been readily available since 2008 with the introduction of the Revised Opioid Prescribing Regulations.^7,75^ Physicians are now able to prescribe opiates for up to 30 days and adjust the dosage according to patients’ needs.^7^ However, opiates are not presently listed among the essential drugs for NCD management at community health stations.^65^ The prevalence of moderate to severe pain among patients with cancer is very high (50% in 2006), and we did not identify any study which assesses the impact of the 2008 revision of Opioid Prescribing Regulations for cancer pain.^76^ Another author also suggested that the development of comprehensive programs, which simultaneously address insomnia, dyspnea, and cough, could be a viable approach to fatigue in patients with cancer.^77^

### National Policies Relating to Cancer Control

The NCCP^9^ was one of 5 programs within the national strategy for the prevention and control of NCDs.^10^ During 2012 to 2015, the cancer control plan aimed to raise community awareness on both the prevention and early detection of cancer, increase the number of early diagnosed cancer cases, and reduce mortality rates of breast, cervical, oral, and colorectal cancer.^9,10^ However, as of 2015, community awareness did not improve, and 79% of patients were still diagnosed at late stages of cancer.^10^ There were also insignificant quantities of data to properly assess the changes in the mortality rate.^10^ The new national strategy for the prevention and control of NCDs during the years 2015 to 2025 set several specific objectives to control the growing cancer burden in Vietnam. These goals include diagnosing 40% of individuals with common cancers at an earlier stage and reaching a 20% relative reduction in premature (aged <70) mortality rate due to cancer, cardiovascular diseases, diabetes, and chronic obstructive pulmonary disease compared to 2015.^10^ A specific plan for cervical cancer prevention and control, which is being implemented between 2016 and 2025, was also approved.^37^ Moreover, during 2012 to 2018, the Ministry of Health also developed 3 national guidelines for the diagnosis and treatment of non-small cell lung cancers, colorectal cancer, and liver cancer.^72^

Although HBV was included in the national vaccination program, there is no plan at the present to adopt and cover the HPV in the vaccination program or the cervical cancer prevention and control action plan of 2016 to 2025.^10,37^ Several studies showed that HPV vaccination against types 16 and 18 would be cost-effective for Vietnam if the cost per individual was less than US$25.^78,79^ Vaccination combined with cervical cancer screening, in this case, would be the most favorable program for Vietnam.^78^ However, if the cost for each vaccination per person is greater than 100 USD, cervical cancer screening alone would be the most cost-effective solution for Vietnam.^78^

In reference to financing cancer care in Vietnam, 80% of cancer services costs (examination and treatment) were covered for most patients with health insurance, and the remaining costs were paid out of pocket.^80^ However, only 50% of the costs for certain expensive or specialized drugs, such as trastuzumab, was reimbursed.^80^ National health insurance also did not cover many essential preventive services, such as tobacco cessation counseling, nutritional counseling, and cancer screening.^65^ Moreover, NCDs programs, including programs for cancer, only received 2.5% to 3.5% of the total national health annual spending, and funding for these health-targeted programs was declining despite their emerging burden.^10,52^

## Discussion

Our review demonstrates that data regarding cancer burden and control in Vietnam are still deficient. Most of the studies we reviewed had nominal sample sizes and were localized, which decreased the generalizability of the presented results. Nevertheless, there was no properly designed population-based cohort which reports data on cancer in Vietnam. Besides some small clinical studies that include patient follow-up, most of the articles were designed as cross-sectional or case–control studies. In general, government investments in population-based cohort studies with multidisciplinary approaches for assessing cancer and other NCDs are genuinely needed. Moreover, the national registry data require significant improvements as only 9 of 63 provinces have their own hospital-based or provincial registries.^7^ Therefore, the quality of incidence and prevalence estimation was relatively poor. In addition, the current registry does not account for or provide any data on cancer survivorship, mortality, and quality of life.^7,16^ The WHO and GICR in tandem have recommended that Vietnam should choose between developing additional cancer incidence registries or bolstering the quality of the current sites.^16,71^

Nevertheless, funding for these previously mentioned cancer prevention programs as a part of NCD-targeted program, accounting for only 2.5% to 3.5% of the national health budget, has been in decline during recent years.^10,52^ The lack of funding could hinder the efforts to bolster cancer research and data quality in Vietnam. In a report detailing national cancer control strategies among Asian countries, The Economist Intelligence Unit has emphasized the “need for more and better data, and evidence-based policy”; this statement is essential as few countries in their report possess a well-built cancer registry with mortality data.^81^ Therefore, we believe that without public investment in research, effective data collection, and prevention programs, Vietnam’s policy-level interventions may not be able to deliver the expected results and achieve a powerful impact.

### Burden of Cancer

Our results have suggested that the burden of cancer in Vietnam has been rising rapidly for the past 30 years. The increased incidence and mortality could be partly explained by the growing prevalence of old and new risk factors as well as improvements in data collection of the death and cancer registry. In 2015, the Economist Intelligence Unit estimated that the proportion of deaths attributable to cancer in Vietnam was 18%, which is similar to several countries within the regions such as Thailand (17%) and Malaysia (15%), but this percentage was higher compared to Indonesia (13%), Myanmar (11%), and India (7%).^81^ Moreover, 70% to 80% of cancer cases in Vietnam were diagnosed at either stage 3 or 4,^7,9^ compared to 57% average of the ASEAN region.^82^ However, the ACTION study reported that the 1-year incidence of mortality in Vietnam (25%) appears to be similar to Thailand (25%) and lower than Indonesia (36%), Philippines (36%), and Myanmar (45%),^24^ but the 1-year incidence of financial catastrophe in Vietnam was the highest in the ASEAN region (68%).^24^ Another compelling finding from the ACTION study was that approximately 7.5 million DALYs were lost in the ASEAN region which can be attributed to cancer in 2008.^24,83^ Surprisingly, 1.7 million cancer DALYs were recorded in Vietnam in the same year, which was equivalent to 22.67% of all DALYs lost to cancer in ASEAN^21,22^—one of the highest among nations in ASEAN.^83^ Nevertheless, the results of this study should be carefully interpreted as there were several considerable biases. For example, the variation in the proportion of patients who completed a 1-year follow-up ranged from 28% to 88% (Vietnam: 78%).^24^ As ACTION was one of the few studies that reported the burden of cancer in Vietnam, we emphasize that there is an urgent need for the production of high-quality data and research activities to strengthen Vietnam’s cancer control effort.

### Primary Prevention: Controlling Important Risk Factors

In tandem with a variety of Asian countries in recent years, Vietnam has achieved more than 90% coverage in the universal infant HBV vaccination program,^81,84^ which is one of the “Best Buys” interventions recommended by the WHO.^85^ However, the high prevalence of chronic HBV (68.2%) in the general population is likely to lead to an increase in HBV-related HCC incidence in the upcoming years,^35^ as HCC may also be driven by hepatitis C, liver fluke, and alcohol drinking. A similar situation can be observed in South Korea where HBV vaccination has been implemented since 1985, but the incidence of HCC is still twice as high as compared to the global average.^81^ We hope that maintaining the coverage for HBV vaccine would help to decrease HCC burden as demonstrated in Taiwan and other similar countries.^81^ Another promising target for primary prevention would be HPV vaccination since it has been observed that nearly 100% of cases of cervical cancer were attributed to HPV infection.^86^ The most common types of HPV in Vietnam were HPV 16 and HPV 18, which could be prevented effectively by vaccination.^39-44^ However, the program would only be cost-effective if the costs per individual was less than US$25,^78,79^ which is a significant financial obstacle for Vietnam. In the ASEAN region besides Malaysia and Singapore, no countries have implemented a national HPV vaccination program,^81,87^ Even Thailand, a country with a relatively good NCCP, still considered HPV vaccination as not cost-effective at this point.^81^ At present, there are 2 possible solutions for Vietnam and other developing countries: (1) negotiate and purchase HPV vaccines at a competitive price from the GAVI Alliance and/or (2) wait until a cheaper generic version of the vaccine is produced and available in the market.

Vietnam should also prioritize on controlling behavioral risk factors, such as tobacco use, alcohol drinking, unhealthy diets, and physical inactivity,^85^ all of which are prevalent throughout Vietnam.^51,52,57^ Some studies in Vietnam revealed the association between these risk factors with breast cancer^53,54^ and prostate cancer^55^; however, these findings were limited by both the sample size and study design. The lack of convincing evidence may slow down the policy development process in Vietnam. It is known that industries could make use of the lack of empirical evidence in the country to push back against many policy initiatives.^88,89^ Regarding Vietnam’s options, the WHO has identified a set of cost-effective and feasible NCDs “Best Buys,” such as tax increases for tobacco and alcohol and increasing public awareness on the benefits of healthy diets and physical activity. Among that list, the most significant issue for Vietnam was tobacco. As our review illustrated, approximately 50% of males in Vietnam are smokers, and the reduction in smoking prevalence was insignificant in recent years.^51^ In the region, the progress also seemed to weaken from 2006 to 2012 as the decline in smoking rates in many countries was lower than anticipated, and the smoking prevalence was shown to have increased in China, Thailand, and Indonesia.^81^ Furthermore, rapid economic development has led to a modification in the pattern of dietary habits, and the population has shifted toward a Western lifestyle, which includes decreased physical activity, in Vietnam and many ASEAN countries.^52,57,81^ However, the evidence of an effective intervention is still lacking. To summarize, we believe that without a firm commitment to the “Best Buys” strategy and effective public health solutions, Vietnam may not be able to curve the rising tide of behavioral risk factors in the population.

Finally, pollution has become a looming problem in Vietnam and Asian countries as a result of rapid development. In addition, there have been reports of numerous so-called “cancer villages” in Vietnam, China, and other countries.^81^ However, we were able to only identify 2 case–control studies with proper design and statistical power, which assessed blood cadmium levels and organophosphate pesticides as potential risk factors for cancer.^59,60^ The other 2 studies, which investigated organochlorines, were all conducted in the early 1990s and suffered from small sample sizes in addition to information bias since the authors did not employ a subjective measurement for the exposures.^61,62^ This situation again stresses the necessity for a proper mechanism from the government to fund a prospective cohort study in the future.

### Secondary Prevention: Improving Screening and Early Detection

Although breast and cervical cancer screenings are recommended to Vietnam by the WHO,^71,85^ our review indicates that there is no national screening program implemented in Vietnam. While many countries in Asia, such as Taiwan, Singapore, Thailand, and China, have implemented their population cancer screening programs,^90^ the pilot screening programs for cervical, breast, oral, and colorectal cancers in Vietnam were only able to reach 2% of the target population.^7,8^ The feasibility of all cancer screening programs mentioned above also requires further evidence to prove their efficacy as only one study reported that the combination of VIA and cryotherapy could be a feasible approach toward cervical cancer screening in Vietnam.^69^ In the near future, low-cost HPV DNA testing in addition to self-sampled vaginal specimens could be another promising solution for Vietnam and many other developing countries.^91^

The major drawbacks to secondary prevention were the organization and funding mechanisms required for maintaining the screening programs. First of all, the programs were centrally operated by health-care providers who work at central/provincial hospitals,^7^ while the WHO recommends the screening procedure to be included as a part of the primary health-care package.^71^ Moreover, there is no centralized registry at the time of this review that allowed easy tracking and follow-up of individuals with positive screening results. This inefficiency prevents screening programs from broadening coverage within the population. Second, cancer screening and other preventive NCDs services, in general, are not covered by the national health insurance system.^52,65^ The lack of insurance coverage could be one of the primary reasons for the low coverage of cervical cancer screening.^57,66^ In a recommendation provided for the Vietnam NCD prevention and control program, the WHO stated that the Vietnamese government should review and implement a mechanism that allows national health insurance to cover essential cancer prevention services at primary health-care centers.^71^ Such funding would also help to relieve the shortage of trained personnel and diagnostic equipment that hinders pilot screening programs.^65^

### Tertiary Prevention: Scaling Up Cancer Care Services

When conducting this review, it was challenging to accurately capture a clear picture of the effectiveness of cancer tertiary care in Vietnam. This difficulty arises as official reports on the medical practice of health professionals, survivorship, in addition to patient satisfaction and quality of life, were found to be absent in the literature. Thus, the cost-effectiveness of a range of interventions would be taxing to assess and interpret in the long run.

In Vietnam, the government is obligated to reserve 30% of the annual health budget for preventive medicine, while the remaining 70% of the budget are supposed to be directed toward tertiary care.^92,93^ With such an enormous investment, cancer care in Vietnam has experienced tremendous improvement during the past few years as the number of radiotherapy units and the oncology departments increased rapidly throughout the country.^7,8,15^ However, such a significant emphasis on cancer treatment in Vietnam, Myanmar, and Malaysia was considered to be not cost-effective in the long term. This is due to the fact that tertiary care facilities would become quickly overwhelmed with late-stage patients while prevention and screening programs are still poorly developed.^81^ Although the national comprehensive cancer centers are very well equipped, numerous provinces in Vietnam still lack a functional cancer service center (10 of 63), pathology departments (9t of 63), and radiotherapy units.^7,15^ Primary health-care centers also lack the required number of health staff with sufficient oncology knowledge and skills to maintain such facilities.^65^ The Ministry of Health even reported that the capacity of the current system could only accommodate for 30% to 40% of the cancer service needs in Vietnam.^65^ This dilemma illustrates that the Vietnamese government is currently facing a difficult challenge, as coverage for curative services must be improved while preventive services also require increased investment. However, pursuing expensive curative care investments is not a cost-effective or long-term solution for cancer care.^81^ Vietnam should at least maintain or even extend the preventive medicine budget beyond 30% of the annual health spending because many of the preventive measures at present were found to be significantly cost-effective and considered to be “Best Buys” by the WHO.^85,94^

Regarding cancer outcomes, 70% to 80% of Vietnamese patients with cancer appeared for treatment at late stages of the illness^7,8^ and experienced poor survival rates.^24-26^ In addition, the coverage of palliative care was found to be severely inadequate in Vietnam.^7^ Our review has also illustrated that palliative care was a component that lagged behind many other areas of NCCP in Asia,^81^ even for developed countries, such as Japan and South Korea.^90^ Moreover, palliative care services in Vietnam are organized in larger hospitals located in urban areas.^7^ In contrast, more than 90% of Vietnamese patients prefer to die at home as they wish to spend their last hours with loved ones in a familiar place.^7^ Therefore, the Vietnam National Cancer Institute is in the process of developing a community-based palliative care model by providing training for community health centers and provincial hospitals, especially for morphine uses and psychosocial support.^7^ One of the bottlenecks for this approach was the lack of opioids and other essential drugs for cancer/NCDs management at community health stations.^65^ Therefore, together with the improvement in the knowledge and skill sets required for health providers at the local level, we also recommend that access to pain relief and other essential drugs at community health stations must be improved in order to create a successful community-based palliative care model in Vietnam.

Last but not least, Vietnamese patients with cancer experienced not only poor outcomes but also the highest rate of financial catastrophe in the ASEAN region.^24^ Although around 80% of cancer services were covered by health insurance, high treatment costs pushed 37.4% of the households with patients with cancer into poverty.^29^ This discrepancy could be partly explained by the high out-of-pocket payment as health insurance only covered 50% of the costs for certain expensive and specialized drugs, which were required for effective cancer treatment.^80^ To improve social security for patients with cancer, the Ministry of Health and national health insurance agencies should conduct a comprehensive health economic analysis to recalculate the necessary payment for cancer services. The policymakers should also explore alternative options to increase the pooling of fund for health insurance by raising taxes on harmful substances, such as tobacco, alcohol, sugar-sweetened beverages, and so forth.

### National Policy: Toward a Better Cancer Control Program

Overall, our review demonstrates that Vietnam is lagging behind in the fight against cancer. The NCCP has been implemented in Vietnam for the past 20 years, but the key indicators of the NCCP show that the results are limited: Cancer awareness stays relatively the same, late stages diagnosis of cancer is still dominant, and there is presently no national mortality data available.^10^ A significant reason for these unfortunate results is the lack of public investment in the program. Our review found that the funding for NCCP together with 4 other NCDs programs was declining and only accounted for less than 3.5% of government health budget.^10,52^ Therefore, the NCCP plan may appear to be well written on paper but ultimately remains impractical due to funding constraints.^81^ This situation was not only limited to Vietnam as many countries in the region, including Myanmar, Malaysia, and Indonesia, also did not have costings and provision of the national budget to support NCCP.^81^

Wealthy countries have more resources at their disposal to control cancer, so their NCCPs are more successful compared to other nations with low resources.^81^ However, how resources are deployed and how the programs are managed also impact the outcomes of NCCPs, even among developed countries.^81^ A prime example for Vietnam would be Thailand, where the comprehensive NCCP with limited resources achieved far better outcomes in comparison to countries with a higher gross domestic product, such as China and Malaysia.^81^ First, Thailand had 16 provincial cancer registries and aimed to cover every province in the near future.^90^ The analysis of cancer data was the foundation for all cancer control policies, which reflected the comprehensive and evidence-based approach of Thai legislators.^81^ Second, Thailand’s NCCP provided the foundation for all services across the cancer control continuum rather than focusing on treatment; the NCCP also emphasizes on cancer data collecting and capacity building.^81,95^ Finally, the Thailand Ministry of Health was also involved with other ministries and government agencies in the process for developing health policies as well as working with/providing funding for nongovernmental organizations, especially to raise the awareness of cancer control in the community.^81,95^ All of these 3 approaches were in line with the Economist Intelligence Unit’s recommendations regarding cancer control plans in Asia: “A need for more and better data, and evidence-based policy, “A need for a more holistic approach to cancer care,” and “A need to engage more with those outside the health system.”^81^ The final recommendation for NCCPs in Asia and Vietnam, in particular, was “A need to consider appropriate legal foundations,” which could provide stable budgets to leverage the requirements for data collection and usage through the country.^81^

### Limitations

Our review comes with several limitations. First of all, because there is no central database for domestic scientific studies, our review did not have the capacity to cover all articles which were published in Vietnamese. Second, many Vietnamese gray literature documents were not in electronic form and could not be found via our online search. Therefore, our ability to create a comprehensive overview of cancer burden and control effort in Vietnam could be limited.

## Conclusion

Given the summary of evidence in this review, it is conclusive that cancer and its associated consequences are both persistent and emerging problem in Vietnam. In addition, the NCCP results are also limited regarding crucial indicators of success. The current situation requires urgent evidence-based control policies on both prevention and the continuity of care for patients with cancer. A holistic approach to cancer services with the participation of various stakeholders, which could be more complicated than working within the health system alone, would be the best direction for Vietnam.

Serious and transparent public investment in data collection and capacity building is needed as high-quality data and further research findings are essential to provide comprehensive insights into the complex situation of cancer in Vietnam.

## Supplemental Material

Supplemental_Material - Cancers in Vietnam—Burden and Control Efforts: A Narrative Scoping Review Click here for additional data file. Supplemental_Material for Cancers in Vietnam—Burden and Control Efforts: A Narrative Scoping Review by Tung Pham, Linh Bui, Giang Kim, Dong Hoang, Thuan Tran and Minh Hoang in Cancer Control
